# Leading the Understanding of Lymphatic Function

**DOI:** 10.1093/function/zqaf038

**Published:** 2025-08-13

**Authors:** Walter L Murfee, Jerome W Breslin, Brant E Isakson

**Affiliations:** J. Crayton Pruitt Family Department of Biomedical Engineering, University of Florida, Gainesville, FL 32611, USA; Department of Molecular Pharmacology and Physiology, University of South Florida, Tampa, FL 33612, USA; Department of Molecular Physiology and Biological Physics, University of Virginia, Charlottesville, VA 22908, USA

## A Perspective on “Hierarchical Requirement for Endothelial Cell Connexins Cx37, Cx47, Cx43 and Cx45 in Lymphatic Valve Function”

Over the past 25 years, interest in understanding the lymphatic system has re-emerged as opportunities for mechanistic insights have been made possible by the expansion of novel vascular biology methods.^1, 2^ The growing field has been marked by the importance of preserving lymphatic function in cancer treatments, the discovery of lymphatic vessels in anatomic sites previously thought to have no lymphatic drainage, identification of critical molecular drivers in lymphatic development, and the creation of journals that specialize in publication of lymphatic research.

Despite the growth in lymphatic research, the major driving questions have largely remained the same with studies focused on (1) development of the lymphatic system; (2) formation of lymph in initial lymphatics (also called lymphatic capillaries); (3) pump function, particularly the lymphatic smooth muscle cell contractile mechanism; (4) lymphatic valve formation, maintenance, and function; (5) remodeling of lymphatic networks, including lymphangiogenesis; (6) entry and trafficking of immune cells, such as dendritic cells; and (7) the genomics and transcriptomics underlying lymphatic diseases. While each of these areas has undoubtably benefited from advances in genetically modified mice and their availability, the field has mostly drifted away from the reductionist approaches that can be used to define physiological function. With most investigations being focused on lymphatic vessel architecture and development of gross edema used to define function, our fundamental understanding of how lymphatic function directly correlates with cellular/molecular mechanisms has lagged the expansion of the field. Physiological views remain essential for appreciating how tissue-specific genetic knockouts are linked to vessel and system level function.

**Figure 1.**
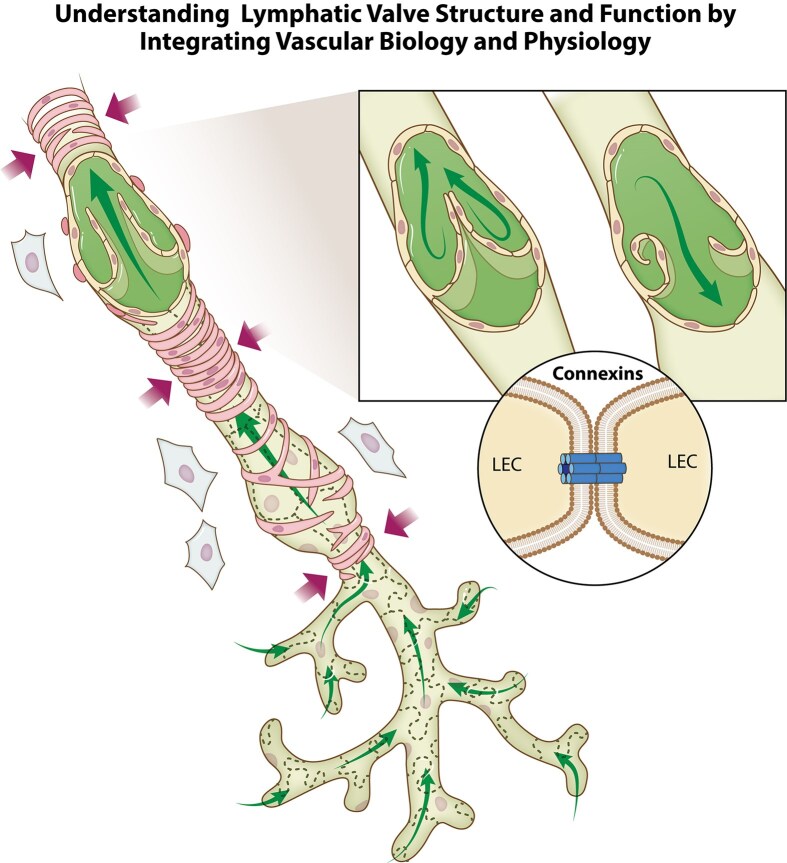
Understanding lymphatic valve structure and function by integrating vascular biology and physiology. Lymphatic system function incorporates fluid transport into initial lymphatic vessels (ie, capillaries) and upstream through contractile vessels, which are wrapped by smooth muscle cells.^1^ The active passing of lymphatic flow along sections of the larger vessels is regulated by multiple-cell interactions culminating in coordinated smooth muscle cell contraction to propel lymph forward and intraluminal endothelial cell valves that prevent flow backward. As highlighted by the work of Davis et al. in the current issue of *Function*, the integration of cell-specific transgenic models and technically challenging measurements of flow and pressures during isolated vessel preparations offer valuable insights for defining the role of connexin isotypes in valve structure and function. Printed with permission from Anita Impagliazzo.

In the current issue of the *Function*, work by Davis et al. integrates technically challenging biomechanical measurements of pressure and flow, isolated vessel preparation, cell-specific gene knockout and gene mutant mouse models, and descriptive structural analysis to characterize lymphatic valve function and the role of different connexin isotypes (Figure 1).^3^ Connexins (Cx) are a family of proteins with over 20 different isoforms; in the blood and lymphatic vasculature, there are at least 5 different isoforms, including Cx37, Cx40, Cx43, Cx45, and Cx47.^4^ The connexins oligomerize to form heteromeric connexons that dock with complementary connexons in adjacent cells to form gap junction channels, directly linking the cytoplasm of one cell to the adjacent cell. This allows the cells in a tissue to synchronize their activity and act as a syncytium. The study by Davis et al. comprehensively evaluates the progression of lymphatic valve defects associated with connexin deficiency and effects on lymphatic flow. Using functional classifications based on measurements of leaflet symmetry, leaflet length, gap length, and pressure differences across the valves, the results provoke new questions about how valves work and the potential implications of valve structure heterogeneity on valves in series functioning adequately. A unique aspect of the study is the application of single, double, and triple knockouts of specific connexin genes to evaluate the roles for different connexin subunits. Identification of a hierarchy for connexin contributions emphasizes an underappreciated sensitivity of physiological function on specific protein types within a family. The findings also provoke consideration of whether the mechanisms involve endothelial cell-cell communication, alternative modes of actions, and whether the field has sufficient appreciation of the spatial organization of endothelial cells along valve leaflets. The study’s meticulous approach and the experimental design to rigorously test the roles of connexin family proteins in lymphatic valve function are thematically in line with *Function*’s aims, scope, and original premise.^5^ As such, the descriptions and findings offer elegant examples for unraveling complex mechanisms that link molecular signaling, cellular coordination, and physiological function.
